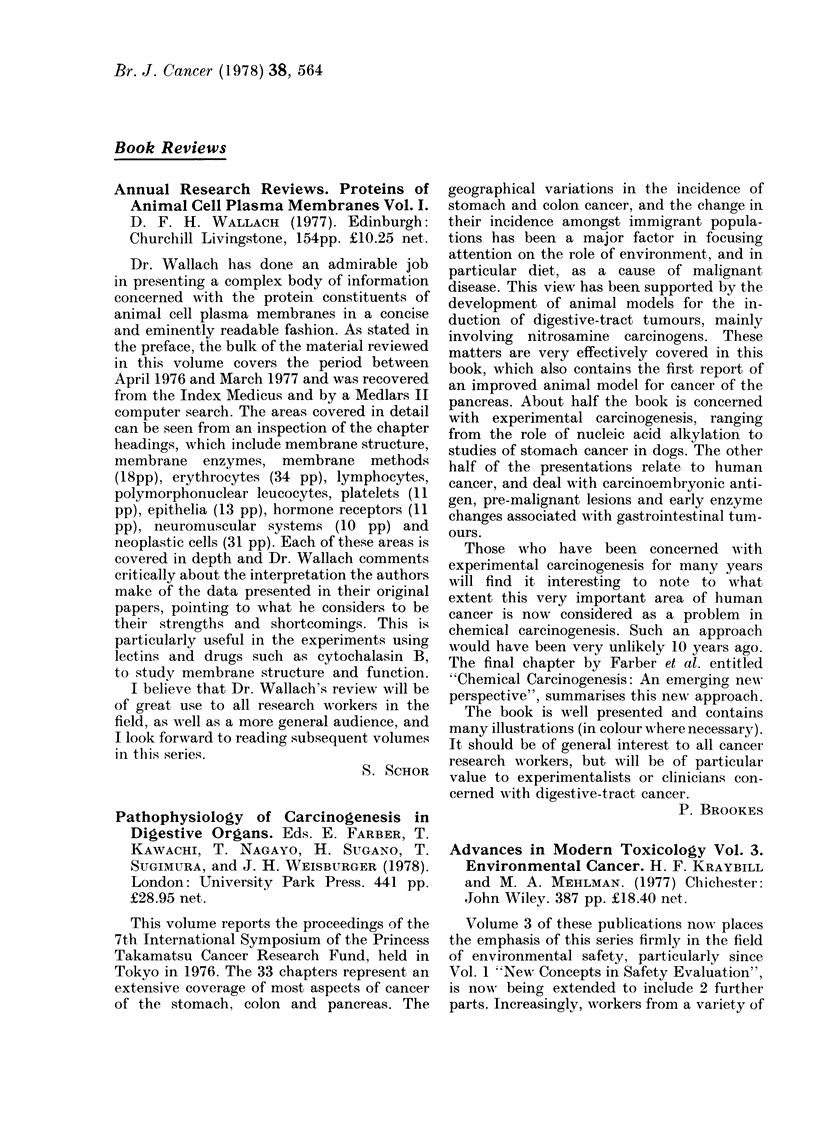# Pathophysiology of Carcinogenesis in Digestive Organs

**Published:** 1978-10

**Authors:** P. Brookes


**Pathophysiology of Carcinogenesis in Digestive Organs.** Eds. E. FARBER, T. KAWACHI, T. NAGAYO, H. SUGANO, T. SUGIMURA, and J. H. WEISBURGER (1978). London: University Park Press. 441 pp. £28.95 net.

This volume reports the proceedings of the 7th International Symposium of the Princess Takamatsu Cancer Research Fund, held in Tokyo in 1976. The 33 chapters represent an extensive coverage of most aspects of cancer of the stomach, colon and pancreas. The geographical variations in the incidence of stomach and colon cancer, and the change in their incidence amongst immigrant populations has been a major factor in focusing attention on the role of environment, and in particular diet, as a cause of malignant disease. This view has been supported by the development of animal models for the induction of digestive-tract tumours, mainly involving nitrosamine carcinogens. These matters are very effectively covered in this book, which also contains the first report of an improved animal model for cancer of the pancreas. About half the book is concerned with experimental carcinogenesis, ranging from the role of nucleic acid alkylation to studies of stomach cancer in dogs. The other half of the presentations relate to human cancer, and deal with carcinoembryonic antigen, pre-malignant lesions and early enzyme changes associated with gastrointestinal tumours.

Those who have been concerned with experimental carcinogenesis for many years will find it interesting to note to what extent this very important area of human cancer is now considered as a problem in chemical carcinogenesis. Such an approach would have been very unlikely 10 years ago. The final chapter by Farber *et al.* entitled "Chemical Carcinogenesis: An emerging new perspective", summarises this new approach.

The book is well presented and contains many illustrations (in colour where necessary). It should be of general interest to all cancer research workers, but will be of particular value to experimentalists or clinicians concerned with digestive-tract cancer.

P. BROOKES